# Pre-treatment Evaluation of Patients Eligible for Whole Brain Radiation Therapy: The Risk of Hippocampal Metastases in a Retrospective Study of 248 Cases at a Single Institution

**DOI:** 10.7759/cureus.49170

**Published:** 2023-11-21

**Authors:** Yojiro Ishikawa, Rei Umezawa, Takaya Yamamoto, Noriyoshi Takahashi, Kazuya Takeda, Yu Suzuki, Keita Kishida, Satoshi Teramura, Kengo Ito, Keiichi Jingu

**Affiliations:** 1 Division of Radiology, Tohoku Medical and Pharmaceutical University, Sendai, JPN; 2 Department of Radiation Oncology, Tohoku University Graduate School of Medicine, Sendai, JPN; 3 Department of Radiation Oncology, South Miyagi Medical Center, Ogawara, JPN

**Keywords:** cingulate gyrus metastasis, cingulum gyrus metastasis, brain metastasis, limbic, hippocampal sparing, whole brain radiation, amygdalar

## Abstract

Whole brain radiation therapy (WBRT) is effective for multiple brain metastases (BMs) but may impair neurocognitive function (NCF). The incidence of hippocampal metastasis (HM) is low, and the factors associated with the occurrence of HM remain unclear. This study aimed to assess the occurrence of limbic system metastasis (LSM), including HM, and to analyze the risk of HM.

We retrospectively analyzed 248 patients who underwent three-dimensional conformal radiation therapy for BMs between May 2008 and October 2015. Gadolinium-enhanced brain MRI or CT scans were used for diagnosis. Statistical analysis involved assessing clinical factors, including age, gender, primary tumor, number of BMs, and maximum metastasis diameter, in relation to the presence of HMs using logistic regression and receiver operating characteristic (ROC) curve analysis.

The median age at treatment was 62 years (range: 11-83 years). Primary lesion sites included the lung (n = 150; 60.5%), breast (n = 45; 18.1%), gastrointestinal tract (n = 18; 7.3%), and bone and soft tissue (n = 2; 0.8%). Histological cancer types included adenocarcinoma (n = 113; 45.6%), squamous cell carcinoma (n = 26; 10.5%), small cell carcinoma (n = 28; 11.3%), invasive ductal carcinoma (n = 35; 14.1%), sarcoma (n = 3; 1.2%), and others (n = 43; 17.3%). MRI or CT scans of the 248 patients were analyzed, indicating a total count of 2,163 brain metastases (median: five metastases per patient). HMs were identified in 18 (7.3%) patients. The most common location for LSMs was the cingulum/cingulate gyrus in 26 (10.5%) patients. In univariate and multivariate analyses, patients with 15 or fewer BMs had a significantly lower incidence of HMs (odds ratio (OR), 0.018 (95% confidence interval (CI), 0.030-0.24)) (p < 0.0001). A maximal tumor size of less than 2 cm significantly increased the incidence of HMs (OR, 13.8 (95%CI, 1.80-105.3)) (p = 0.0003). The presence of cingulum/cingulate gyrus metastases also demonstrated a significant increase in the incidence of HMs (OR, 9.42 (95%CI, 3.30-26.84)) (p < 0.0001).

The present study has uncovered a novel association between a high number of metastases in the cingulate gyrus and the development of HMs. Patients with BMs eligible for WBRT with metastases in the cingulate gyrus may be at risk of developing HM.

## Introduction

Metastasis is one of the primary causes of death among cancer patients [1]. Historically, the prognosis of patients with brain metastases (BMs) is unfavorable. In the recursive partitioning analysis, the survival range was 2.3-7.1 months based on severity. The diagnosis-specific graded prognostic assessment considers primary tumor type and features; the survival range was 2.79-25.30 months [2], with an average life expectancy of approximately one month without intervention [3]. Managing BMs is of paramount significance for cancer patients, given that research suggests that 10%-40% of individuals with cancer ultimately experience the development of BMs [4,5].

Whole brain radiation therapy (WBRT) is an effective treatment for multiple BMs in adults [6,7]. The delivery of chemotherapy to the brain is limited by the blood-brain barrier, which can render it ineffective [8]. It may also restrict its usage due to side effects. WBRT can treat the entire brain, including the cerebral hemisphere, cerebellum, limbic system (LS), and brain stem. Although its use is decreasing with the advent of newer techniques such as stereotactic radiosurgery (SRS), the superiority of SRS to WBRT, especially for patients with a solitary or limited number of metastatic brain lesions, is unclear.

However, neurocognitive function (NCF) impairment is a common late toxicity following WBRT, causing decreased quality of life for patients [9,10]. To decrease the risk of NCF impairment, sparing the hippocampus during WBRT has been recommended [11,12].

Hippocampal metastasis (HM) alone is infrequent and more likely to occur in conjunction with metastatic lesions in other parts of the brain. Therefore, in previous studies, the risk assessment of HM has often involved comparing the number or volume of tumors in other parts of the brain, in addition to the hippocampus [11,13-15]. Some recent reports have attempted to classify risk based on the distance from the hippocampus. However, measuring and generalizing this distance can be challenging [16]. Hence, we directed our attention to limbic system metastases (LSMs), which are embryologically and anatomically close to the hippocampus [11,17]. Given the proximity of the LS to the hippocampus regarding blood flow, we hypothesized that an increased risk of metastasis in the LS would also elevate the risk of metastasis to the hippocampus.

The main objective of this study was to determine the occurrence of LSM and HM based on embryological and anatomical structures. This investigation aimed to enhance the comprehensive assessment of HM risk. Our goal was to establish a connection between the risk of metastasis in the limbic system (LS) and the risk of metastasis in the hippocampus. This would provide foundational data for future phase II and phase III trials involving WBRT, emphasizing the need for preserving the hippocampus.

## Materials and methods

This retrospective study was conducted in accordance with the Declaration of Helsinki and received approval from the Tohoku University Graduate School of Medicine Ethics Committee (approval number: 2016-1-070). The study involved patients who underwent three-dimensional conformal radiation therapy for brain metastases (BMs). Among the 260 patients with BMs treated between May 2008 and October 2015 with whole brain radiotherapy (WBRT), those with lesions that could not be measured and 12 patients with only dural or leptomeningeal metastases were excluded, resulting in 248 patients with BMs included in the analysis.

Prior to WBRT, each patient underwent a gadolinium-enhanced MRI or CT scan. The selection of diagnostic images and the method of capturing each diagnostic image were at the discretion of the attending physician. BMs were detected solely through CT scans in eight cases. We utilized an MRI with a 1.5 T MRI system, which included axial T1-weighted spin-echo, axial T2-weighted spin-echo, and axial gadolinium-enhanced T1-weighted spin-echo sequences. For each BM diagnosed by one or two experienced radiologists, one radiation oncologist conducted the tumor counting and measurements necessary for the current analysis.

**Figure 1:**
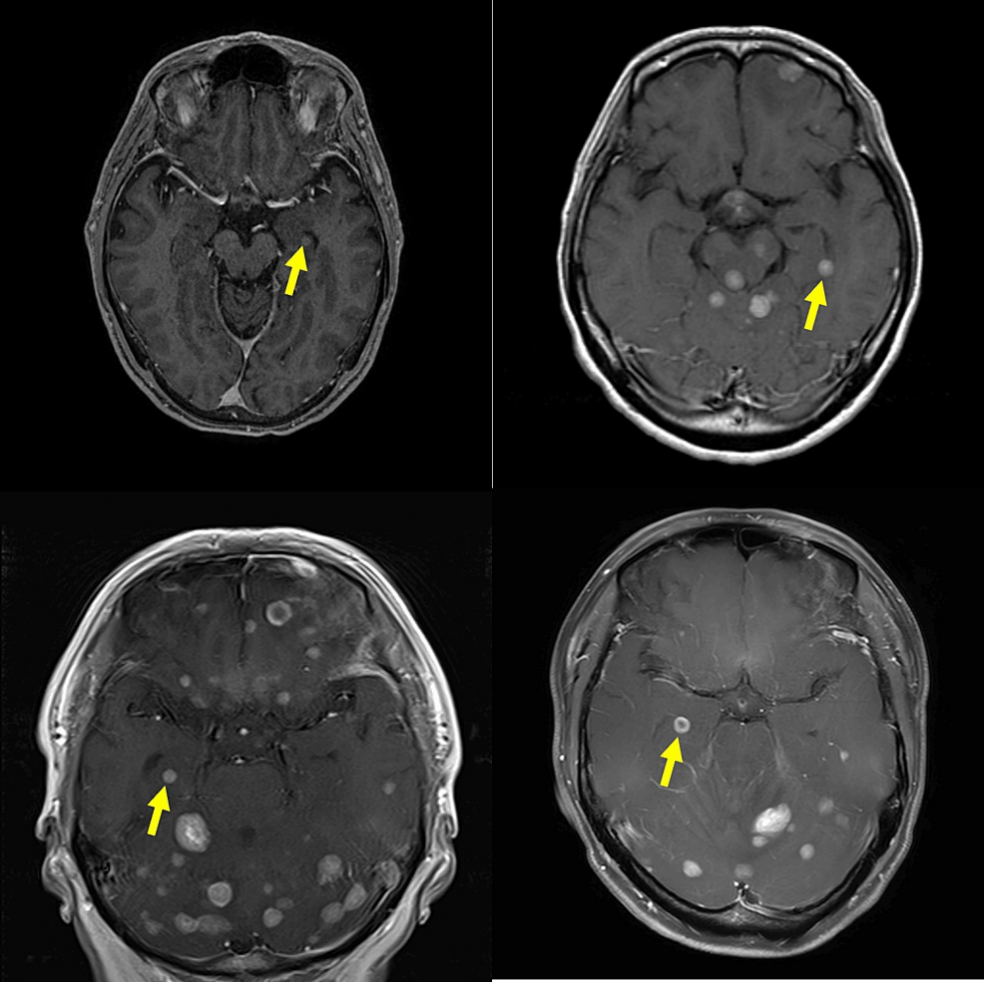
Example of hippocampal metastases The arrow points to the hippocampal metastasis.

**Figure 2:**
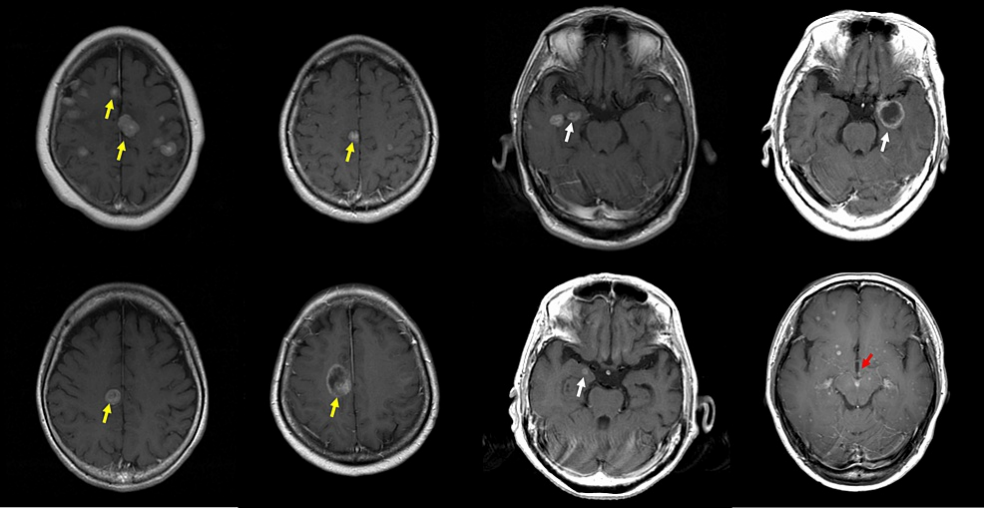
Example of limbic system metastases Yellow, white, and red arrows indicate examples of cingulate gyrus metastasis, amygdala metastasis, and mammillary body metastasis, respectively.

The prevalence of BM in the hippocampus and its surrounding region, known as the LS, was calculated. The anatomical boundaries of the hippocampus were defined in accordance with the standards established in the Radiation Therapy Oncology Group (RTOG) 0933 [18]. Differences in the prevalence of HM based on tumor characteristics and distribution within the limbic region were analyzed. Examples of HM are shown in Figure 1, and examples of LSM in the amygdala, cingulate gyrus, and mammillary body are shown in Figure 2.

Statistical analysis

Categorical variables were compared using the χ2 test or Fisher's exact test, and continuous data were compared using the Mann-Whitney U test. Logistic regression was performed to analyze the correlation between HMs and various factors such as age, gender, primary tumor, pathological type, number of BMs including BMs and LSMs, and maximum diameter of metastases including BMs and LSMs. The optimal cutoff values for the number of BMs and the maximum diameter of metastases were obtained by calculating the area under the receiver operating characteristic (AUC-ROC) curve. Statistical analyses were performed using JMP version 17 (SAS Institute Inc., Cary, NC). Statistical significance was defined as a value of p < 0.05 in the present study. In all analyses, a p-value of <0.05 was considered statistically significant.

## Results

The median age at treatment was 62 years (range: 11-83 years). A total of 110 (44.3%) patients had a Karnofsky Performance Status (KPS) score of 70 or greater. Primary lesion sites included the lung (n = 150; 60.5%), breast (n = 45; 18.1%), gastrointestinal tract (n = 18; 7.3%), bone and soft tissue (n = 2; 0.8%), malignant lymphoma (n = 4; 0.2%), uterus (n = 3; 1.2%), ovary (n = 3; 1.2%), kidney (n = 2; 0.8%), testicle (n = 2; 0.8%), prostate (n = 2; 0.8%), bladder (n = 1; 0.4%), thyroid (n = 1; 0.4%), thymus (n = 1; 0.4%), pharynx (n = 1; 0.4%), and primary undetermined (n = 1; 0.4%). Histological cancer types included adenocarcinoma (n = 113; 45.6%), squamous cell carcinoma (n = 26; 10.5%), small cell carcinoma (n = 28; 11.3%), invasive ductal carcinoma (n = 35; 14.1%), sarcoma (n = 3; 1.2%), and others (n = 43; 17.3%). The primary tumor was controlled in 25 (10%) patients. Table 1 lists the patient- and tumor-related characteristics in this study.

**Table 1:** Patient and tumor characteristics

	Number or range	% or median
Patients	248	100%
Age (years)	11-83	62 (median)
Male/female	133/115	53.6%/46.4%
Primary lesion site		
Lung	150	60.5%
Breast	45	18.1%
Gastrointestinal	18	7.3%
Uterus	3	1.2%
Bone and soft tissue	2	0.8%
Other	30	12.1%
Histology		
Adenocarcinoma	113	45.6%
Squamous cell carcinoma	26	10.5%
Invasive ductal carcinoma	35	14.1%
Small cell carcinoma	28	11.3%
Sarcoma	3	1.2%
Other	43	17.3%
Number of lesions	1-71	5 (median)
>15	38	15.3%
≤15	210	84.7%
Tumor size (cm)	0.3-6.1	1.68 (median)
>2	144	58%
≤2	104	42%
Primary lesion		
Controlled	25	10%
Uncontrolled	223	90%
Brain surgery		
With	17	6.9%
Without	231	93.1%
Stereotactic radiosurgery		
With	44	17.7%
Without	204	82.3%
Headache		
With	59	23.8%
Without	189	76.2%
Motor/sensation deficit		
With	70	28.2%
Without	178	71.8%
Edema of peripheral brain tumors		
With	139	56%
Without	109	44%

Prevalence of hippocampal and other LSMs

We analyzed the MRI or CT scans of the 248 patients. A total of 2,163 metastases were detected (median: five metastases per patient). The median time from diagnosis of the primary tumor to BM was 13 months. In total, 40 (16.1%) patients had LSMs. HMs were detected in 18 (7.3%) patients. Amygdala metastases (AMs) were detected in five (2%) patients with MRI. The most common location of LSMs was the cingulum/cingulate gyrus for 26 (10.5%) patients. The cases of HM were evenly distributed among both genders, with nine cases each. The median time from diagnosis of the primary tumor to HMs was 12.5 months (range: 0-72 months). All 248 patients were treated with WBRT in this study. Table 2 shows the number of LSMs based on the number of patients, while Table 3 shows the number of LSMs based on the number of BMs.

**Table 2:** Prevalence of limbic system metastases based on the number of patients

	Number of patients	%
Brain metastasis	248	100%
Hippocampal metastasis	18	7.3%
Amygdala metastasis	5	2%
Cingulum/cingulate gyrus metastasis	26	10.5%
Mammillary body metastasis	0	0%
Limbic system metastasis	40	16.1%

**Table 3:** Prevalence of limbic system metastases based on the number of brain metastases

	Number	%
Number of brain metastases	2,163	100%
Hippocampal metastases	18	0.8%
Amygdala metastases	5	0.2%
Cingulum/cingulate gyrus metastases	31	1.4%
Mammillary body metastases	0	0%
Limbic system metastases	53	2.5%

In univariate analysis, patients with BMs of 15 or less than 15 had a significantly decreased incidence of HMs (odds ratio (OR), 0.018 (95% confidence interval (CI), 0.030-0.24)) (p < 0.0001). The AUC value for the cutoff point of brain metastasis count, as determined using the AUC-ROC, was 0.79. A maximal tumor size of less than 2 cm had a significantly increased incidence of HMs (OR, 13.8 (95%CI, 1.80-105.3)) (p = 0.0003). The AUC value for the cutoff point of maximal tumor size, as determined using the AUC-ROC, was 0.60. The presence of cingulum/cingulate gyrus metastases had a significantly increased incidence of HMs (OR, 9.42 (95%CI, 3.30-26.84)) (p < 0.0001). Age, presence of lung cancer, presence of headache, presence of motor/sensation deficits, and presence of metastasis peripheral edema showed no significant differences in univariate analysis. In multivariate analysis, patients with BMs of 15 or less than 15 had a significantly decreased incidence of HMs (OR, 0.11 (95%CI, 0.037-0.35)) (p = 0.0001). A maximal tumor size of less than 2 cm had a significantly increased incidence of HMs (OR, 10.22 (95%CI, 1.23-84.9)) (p = 0.03). The presence of cingulum/cingulate gyrus metastases had a significantly increased incidence of HMs (OR, 6.34 (95%CI, 1.94-20.75)) (p = 0.0023) (Table 4).

**Table 4:** Univariate and multivariate analyses p-value < 0.05 indicates statistical significance. OR: odds ratio, 95%CI: 95% confidence interval

	Univariate		Multivariate	
Parameter	OR (95%CI)	p-value	OR (95%CI)	p-value
Age (years)				
>60	1	-	-	-
≤60	1.07 (0.41-2.83)	0.88	-	-
Histology				
Lung cancer	1	-	-	-
Non-lung cancer	0.75 (0.28-1.96)	0.56	-	-
Number of lesions				
>15	1	-	-	-
≤15	0.08 (0.030-0.24)	<0.0001	0.11 (0.037-0.35)	0.0001
Tumor size (cm)				
≥2	1	-		
<2	13.8 (1.80-105.3)	0.0003	10.22 (1.23-84.9)	0.03
Brain surgery				
Without	1	-	-	-
With	0.79 (0.098-6.30)	0.82	-	-
Stereotactic radiosurgery				
Without	1	-	-	-
With	1.36 (0.42-4.34)	0.62	-	-
Headache				
Without	1	-	-	-
With	0.91 (0.29-2.88)	0.87	-	-
Motor/sensation deficit				
Without	1	-		
With	0.30 (0.0067-1.33)	0.067	-	-
Peripheral edema				
Without	1	-	-	-
With	1.25 (0.47-3.35)	0.65	-	-
Limbic system metastases				
Amygdala metastases				
Without	1	-	-	-
With	3.32 (0.35-31.40)	0.35	-	-
Cingulum/cingulate gyrus metastases				
Without	1	-	-	-
With	9.42 (3.30-26.84)	<0.0001	6.34 (1.94-20.75)	0.0023

## Discussion

This study aimed to explore the connection between the frequency of HM and LS. The pathways and mechanisms of BMs are intricate, with several theories proposed. While BM sites are often uniform, it is acknowledged that metastases can be unevenly distributed depending on the underlying disease and other factors [19,20]. For instance, some studies have suggested a higher incidence of metastasis to the posterior cranial fossa in breast cancer [21].

BMs are thought to be related to the arterial structure, implying a link between artery distribution and BM distribution. The LS shares some vascular similarities with the hippocampus. The hippocampus is supplied by the anterior choroidal artery (AChA) and the main trunk of the posterior cerebral artery (PCA), while the amygdala is primarily supplied by the AChA. The posterior cingulate gyrus is also supplied by the PCA [11,17,22,23]. The LS is an embryologically primitive region with an interconnected network of neural pathways. It is reasonable to posit that HM is related to LSM. No studies have explored the relationship between hippocampal and limbic transitions.

Marsh et al. reviewed 697 intracranial metastases in 107 patients and reported that 36 (5.2%) involved mainly metastatic limbic circuits, 16 (2.29%) were in the hippocampus and 20 (2.86%) in the cingulate gyrus [11], but they did not note amygdala and mammillary body metastases. Amygdala metastases were observed in this study, possibly due to the large number of cases with multiple metastases eligible for WBRT.

The hippocampus is one of the structures of the LS and is physically close to the other structures of LS, including the amygdala, cingulum/cingulate gyrus, and mammillary body, and previous reports also have attempted to identify risk factors based on physical distance from the hippocampus [24]. Ghia et al. reviewed 272 cases of intracranial metastases and reported that 3.3% had metastases within 5 mm of the hippocampus, while 86.4% were more than 15 mm from the hippocampus [14]. Gondi et al. retrospectively studied the distribution of 1,133 metastases from 371 patients. None of the metastases were within the hippocampus. Metastases within 5 mm of the hippocampus were found in 8.6% of patients and 3% of BMs in the entire cohort [15]. Many of the BMs assessed in these studies may have included metastases in the LS, which is closely situated in the hippocampus.

Previous reports have primarily emphasized that the risk of HM would likely increase with a rise in the number and volume of BMs [15,16]. In the present study, patients with more than 15 metastases were also considered to be at increased risk of HMs. Another study has shown that the risk of HMs exists even when the number of metastases is relatively small. Guo et al. also reported that patients with BMs more than or equal to five had a significantly higher risk of HMs [25].

Gondi et al. found that for each cubic centimeter increase in intracranial metastasis, the odds of metastasis to the perihippocampal area increased 1.02-fold (95% confidence interval (CI), 1.006-1.034) [15]. Our results showed some differences in this aspect. In this study, we compiled data on the size of the largest tumor among multiple brain metastases. The findings indicated that metastases measuring less than 2 cm increased the risk of HM. This could be interpreted as suggesting that in this study, there was a higher prevalence of patients with multiple metastases, and HMs were more frequently observed in patients with multiple small nodules measuring less than 2 cm in diameter.

Following the results of the RTOG prospective study [18], hippocampal-sparing whole brain radiation therapy (HS-WBRT) is now administered to patients with BMs. However, cases of HMs have been reported as an outcome of HS-WBRT. Additionally, an increasing number of research papers have raised concerns about the risk of recurrence following HS-WBRT. Harth et al. reported that HS-WBRT with the commonly applied fractionation scheme would increase the risk of brain recurrence by 4% compared to conventional WBRT [26].

This study has several limitations: (i) It was conducted retrospectively, which could introduce bias due to the way medical records were documented and patient information was collected. (ii) It was difficult to distinguish between the hippocampus and the parahippocampal gyrus in MRI [27]. Therefore, the specificity for detecting the incidence of HMs might be low. (iii) The present study only analyzed patients after WBRT. Therefore, patients who were treated with surgery or SRS alone for BMs were not included in this study. Although the study included a sufficient number of cases (248 cases), it should be noted that the focus of this study was not so much on the frequency of HMs in BMs but rather on the frequency of HMs in patients eligible for WBRT. (iv) It was challenging to exclude cases in which a large number of metastases affect the frequency of LMs or HMs. The results of the present study may only indicate that a higher number of brain metastases are associated with the prevalence of LMs or HMs. (v) The small number of diagnosticians involved in the diagnosis must also be added to the limitations of this study. (vi) In this study, some cases were included in which the number of brain metastases was limited (oligo-brain metastasis). WBRT was recommended for these cases, but this decision was influenced by the primary tumor's condition and the patient's treatment preferences. Currently, oligo-brain metastasis cases are typically treated with SRS, so it might have been more appropriate to exclude them from this study, which primarily focuses on assessing the risks associated with HS-WBRT.

## Conclusions

This study was designed to determine whether the risk of hippocampal metastases in patients with multiple brain metastases can be predicted by the development of brain metastases in the limbic system, which is closely related to the hippocampus. As in previous case reports, the risk of hippocampal metastasis was confirmed to increase with the number of brain metastases. The present study has unveiled a new association between a high number of metastases to the cingulate gyrus and the development of hippocampal metastases. Consequently, for patients with LSMs eligible for WBRT, HS-WBRT may be considered as a potentially risky treatment option.

It is important to note that this study was retrospective in nature and confined to cases where WBRT was recommended. Therefore, it is imperative to incorporate other cases with brain metastases in future studies and gather more substantial evidence, particularly through prospective research.
